# Supplementary material for: Programmable base editing of zebrafish genome using a modified CRISPR-Cas9 system
Source: Nat Commun. 2017 Jul 25;8:118. doi: 10.1038/s41467-017-00175-6 (PMC5524635; doi:10.1038/s41467-017-00175-6)
Supplement: Supplementary file 2 — Supplementary Information [file 41467_2017_175_MOESM2_ESM.pdf]

File name: Supplementary Information
Description: Supplementary tables.

File name: Peer review file
Description:

Supplementary Table 1. Target sites in this study. Red colors means PAM sequences.

Gene name	Target sites
twist2-1	gGCTCCAGAACCAGCGCGTCCTGG
twist2-2	gGCCGCTCGCGTACGTTCGCCAGG
twist2-3	GCGTCCTGGCGAACGTACGCGA
gdf6	GGTCGATCAGAGAGGCCACTGG
ntl	GGAACCAGCCACCGACTGTTGG
tyr	GGCAATCCCGGGGACCACGACCGG
urod-1	GAGATCGTGAGGAGGATGTTGGA
urod-2	GTTCCCCTGAAGCTTGCTGTGA
tial1	GGCCTCCGTAACATCCCTGGAGA

Supplementary Table 2. Primers used in this study.

Name	Sequence of the primer (5'-3')
twist2-1 gRNA	TAATACGACTCACTATAgGCTCCAGAACCAGCGCGTCC GTTTTAGAGCTA
twist2-2 gRNA	TAATACGACTCACTATAgGCCGCTCGCGTACGTTCGCC GTTTTAGAGCTA
gdf6 gRNA	TAATACGACTCACTATAgGTCGATCAGAGAGGCCACGTTTTAGAGCTA
ntl gRNA	TAATACGACTCACTATAGGAACCAGCCACCGACTGT GTTTTAGAGCTA
tyr gRNA	TAATACGACTCACTATAgGCAATCCCGGGGACCACGAC GTTTTAGAGCTA
twist2-3 gRNA	TAATACGACTCACTATAgGCGTCCTGGCGAACGTACGGTTTTAGAGCTA
urod-1 gRNA	TAATACGACTCACTATAgGAGATCGTGAGGAGGATGTT GTTTTAGAGCTA
urod-2 gRNA	TAATACGACTCACTATAgGTTCCCCTGAAGCTTGCTG GTTTTAGAGCTA
tial1 gRNA	TAATACGACTCACTATAGGCCTCCGTAACATCCCTGG GTTTTAGAGCTA
tracr rev	AAAAAAAGCACCGACTCGGTGCCAC
twist2 detect	F: AAGTGGAGATCGTATTTTCTC
	R: GTTCGGCATTATCAGGACGCG
gdf6 detect	F: GCTCTCTCCTTTGCGAAGAC
	R: AATCCCCCAAACGTCCAGAA
ntl detect	F: GCCAAAGCTTTCCTCGATGC
	R: TTGCTCTTACTGGTGGTAGTGC
tyr detect	F: TGAAAGTTACAACCTCCGCGA
	R: AGTCAGTGAAAGTCAGGAACA
urod-1 detect	F: CAAATTTAGGCCCTGCTGG
	R: TTAGCGTTTGAGAAGCTGGC
urod-2 detect	F: GACTCCACAAGTCACATGTTC

	R: AATGTAATGACACAGCCGT
tial1 detect	F: CCAGGGATGTTACGGAGGCC
	R: GGCCTCCGTAACATCCCTGG
genotyping for *tyr* locus	F: GCGTCTCACTCTCCTCGACTCTTC
	R: GTAGTTTCCGGCGCACTGGCAG
VQR SpCas9 R1335Q,T1337R double mutation	F: GATAGAAAGcaGTACAgaTCCACTAAGGAGGTGCTGGACGCTACACTGA
	R: CCTTAGTGGAtcTGTACtgCTTTCTATCGATTGTAGTGTCGAAGTACTTG
VQR SpCas9 D1135V	F: GGAGGCTTTGtCTCTCCCACTGTGGCATACTCCGTCCTGGTG
	R: AGTGGGAGAGaCAAAGCCTCCATACTTCTTAGGGTCCCAGTC
H840A mutation	F: CGACGTGGATgcTATCGTCCCCCAGAGTTTCATCAAAGATGAC
	R: GGGGACGATAgcATCCACGTCGTAATCAGACAGCCTGTTAATA
